# Computational Investigation of Interactions between Carbon Nitride Dots and Doxorubicin

**DOI:** 10.3390/molecules28124660

**Published:** 2023-06-09

**Authors:** Mattia Bartoli, Elena Marras, Alberto Tagliaferro

**Affiliations:** 1 Center for Sustainable Future Technologies, Italian Institute of Technology, Via Livorno 60, 10144 Torino, Italy; mattia.bartoli@polito.it; 2 Consorzio Interuniversitario Nazionale per la Scienza e Tecnologia dei Materiali (INSTM), Via G. Giusti 9, 50121 Firenze, Italy; 3 Politecnico di Torino, Department of Applied Science and Technology, C.so Duca Degli Abruzzi 24, 10129 Torino, Italy; elena.marras@studenti.polito.it

**Keywords:** carbon nitride dots, Doxorubicin, modelling

## Abstract

The study of carbon dots is one of the frontiers of materials science due to their great structural and chemical complexity. These issues have slowed down the production of solid models that are able to describe the chemical and physical features of carbon dots. Recently, several studies have started to resolve this challenge by producing the first structural-based interpretation of several kinds of carbon dots, such as graphene and polymeric ones. Furthermore, carbon nitride dot models established their structures as being formed by heptazine and oxidized graphene layers. These advancements allowed us to study their interaction with key bioactive molecules, producing the first computational studies on this matter. In this work, we modelled the structures of carbon nitride dots and their interaction with an anticancer molecule (Doxorubicin) using semi-empirical methods, evaluating both geometrical and energetic parameters.

## 1. Introduction

Since their discovery in 2004 [1], carbon nano dots (CDs) have attracted great interest due to their unique set of properties [2]. CDs are characterized by a high fluorescence emission [3] due to their quantum confinement [4], water solubility [5] and high biocompatibility [6]. Their chemical features have spread their use across several fields with remarkable achievements as fluorescent probes in analytical procedures [7,8,9] and biomedical treatments [10,11] as active materials for both photocatalysis [12,13] and electrocatalysis [14,15]. The application of CDs has raised several issues related to what is and what is not a carbon dot together with serious concern about the development of a rational classification methodology [2].

The classification is of utmost relevance to properly approaching the field of CDs [16]. The most widespread classification approach is based on structural features, and it encompasses three large families of CDs [17]: (i) graphene quantum dots (GQDs), (ii) carbon nitride carbon dots (CNDs) and (iii) polymeric carbon dots (PCDs).

GQDs represent the first kind of CDs discovered and they present the simplest structures among all CDs. The chemical structure of this family is directly related to small graphene oxide clusters, and the distribution of chemical functionalities is explained simply by the Lerf–Klinowsky model [18]. Accordingly, GQDs contain few layers of oxidized graphene, with carbonyls and carboxylic residues being concentrated in the edges, producing an oxygen-rich shell that accounts for their water solubility. The oxidized graphene layers of GQDs display different morphologies and oxidation degrees [19,20] that provide different chemical platforms for further functionalization [21,22].

PCDs are a wide family of nanomaterials produced by the partial degradation of polymers [23] or through the condensation of organic precursors [24,25,26]. Their properties are very difficult to correlate with a unique interpretative model and each PCD requires proper characterization. 

CNDs are similar to GQDs, but they present a more complex structure in which nitrogen atoms dope the graphene layers [27] or form nitrogen-rich aromatic moieties, as found by Mintz et al. [28]. This represents a very interesting possibility for synthetic chemistry, as it can tune the reactivity and properties [29], particularly in a watery medium, as mentioned by Wiśniewski [30]. As a consequence of their functional tuneability, CNDs can act as a platform for several specific tasks, such as chemotherapy for cancer treatment, by being conjugated with active molecules [31]. Among them, Doxorubicin is one of the most studied, due to its remarkable activity, biological stability and simple chemical structure [32,33]. CNDs were successfully combined with Doxorubicin for the production of conjugates (D-CNDs) for the treatment of several cancers using theragnostic approaches [34,35,36]. Even if the interest in the rational design of D-CNDs is great, the literature lacks computational studies devoted to quantifying the interactions between Doxorubicin and CNDs. Rashid et al. [37] investigated flutamide CNDs through DFT calculation, limiting the system to a representative portion of small CNDs. Zaboli et al. [38] went more in depth by simulating the interaction of a molecule of Doxorubicin on a single sheet of carbon nitride, taken as a representative model of CNDs. The authors were able to correctly evaluate the π−π and water interaction with both a single large layer of heptazine and the Doxorubicin-conjugated material. Similarly, Shaki et al. [39] investigated the adsorption of and interaction between several anticancer species with the external and inner surfaces of carbon nanotubes. All of these studies show a common point represented by the charge transfer from the drugs to the aromatic domain-rich carrier and assume that this is the key reason for the efficacy of the systems.

These approaches are powerful, but they require a high calculation power, and they are currently limited to small chemical systems that are not sufficiently complex to properly describe CDs. Alternatively, semi-empirical quantum chemistry has been developed to resolve the computation challenges of complex systems in order to try to overcome the main limitations of the traditional Hartree–Fock approaches, the slow speed and the low accuracy [40]. The semi-empirical methods are based on the time reduction of the calculation by parameterizing or omitting certain terms based on the experimental data sources (i.e., the ionization energies of atoms, dipole moments of molecules) [41]. Accordingly, the semi-empirical quantum chemistry approaches are considerably faster and useful for modelling large molecules. The first semi-empirical quantum chemistry method was the modified neglect of the diatomic overlap (MNDO) [42] that parameterized the one-center two-electron integrals based on the spectroscopic data for the isolated atoms, evaluating other two-electron integrals using the multipole–multipole interactions of the classical electrostatics [43]. The MNDO approach was implemented using a basis set composed of only s and p orbitals even if the d orbitals set was used to describe hypervalent sulfur atoms and transition metals. Nevertheless, MNDO is characterized by several intrinsic limitations, such as the impossibility to describe both hydrogens or to predict the heat of the formation [44]. Dewar and coworkers [45] improved the MNDO approach by developing the Austin Model X (AMX) by modifying the expression of nuclear–nuclear core repulsion, emulating van der Waals interactions. This new approach required a total reparameterization of the dipole moments and ionization potentials but allowed the description of the hydrogen bond network and the heat of the formation without properly estimating the basicity.

All of these approaches were made outdated by the development of parametric method 3 (PM3) [46], which uses a Hamiltonian operator that is very similar to the AM1 Hamiltonian, but the parameterization strategy is one of molecular properties, rather than atomic ones. A consolidating method which can be used to perform the computational modelling of large organic systems with lower computational costs is the PM3. This is a semi-empirical quantum mechanical parameterization method based on the modified neglect of the differential overlap method [47].

The PM3 approach is based on the use of a set of empirical parameters to describe the electronic structure of a molecule [48]. The key feature of the PM3 is that it offers the chance to accurately predict the geometrical conformation of large molecules, including enzymes [49] and polymers [50].

Despite these advantages, the PM3 method does not perform well enough to accurately describe the properties of the transition metal complexes [51] and the effects of electron correlation [52].

In the present work, we report a computational study focused on the evaluation of the geometrical features of D-CNDs and the interaction energies (E_i_) between Doxorubicin and CNDs using a PM3-based computational approach. We ran a semi-empirical simulation of four-layer CNDs with a single molecule of Doxorubicin, evaluating different geometrical interactions with the Doxorubicin of the outer layer of the CNDs and evaluating the intercalant agent in order to provide a first solid insight into the non-covalent interactions occurring within a chemotherapy agent.

## 2. Results

### 2.1. CNDs Model Structure: Definition and Modelling

The most challenging issue in the modelling of CNDs is to provide a representative species to be studied. The structural composition of CNDs is highly dependent on the synthetic method used for their production. CNDs are generally composed of a core composed of sp^2^-hybridized carbons surrounded by a surface layer that contains a mixture of sp^2^-/sp^3^-hybridized carbon and nitrogen atoms [53]. Regarding pure carbon nitride structures, the nitrogen atoms are incorporated directly into their carbon lattice, creating structural defects and altering the electronic properties of the material. Considering CNDs, the distribution of nitrogen atoms is still a matter of discussion, and several hypotheses have been suggested to explain their several spatial arrangements.

**Figure 1:**
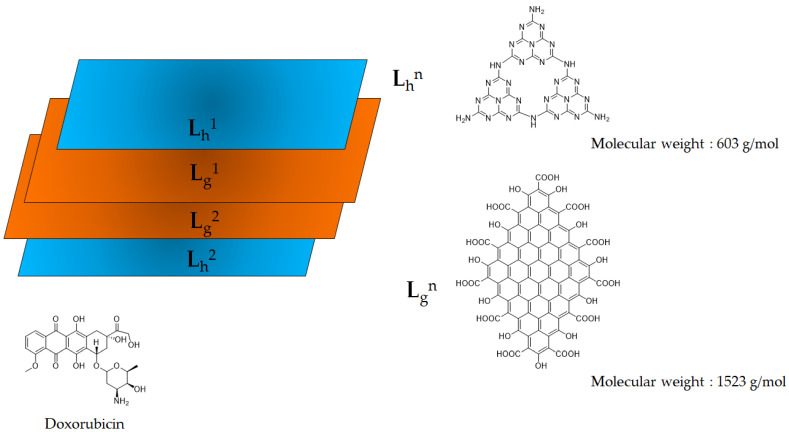
Scheme of representative species used to model Doxorubicin, CNDs and D-CNDs.

Firstly, those conducting CND structural investigations have hypothesized a pure layered structure formed by functionalized heptazine units [27]. Mintz et al. [28] moved a step forward in the clear definition of the structure of CNDs, suggesting a more complex arrangement of the layers. Based on a detailed physical–chemical investigation, the authors proved that CNDs have a graphitic core with massive functionalization on the edges and less heteroatomic inclusion in the core. As reported by Zhou et al. [54], elucidating the differences between a pure heptazine and a realistic model was crucial in order to properly evaluate the interaction between the CNDs and the protein active site. Nevertheless, the great variability of their functionalization prevented the realization of a general model compound that is able to describe all CNDs species. Accordingly, we tentatively propose a model that considers both the heptazine and functionalized graphene layers, as shown in Figure 1.

**Figure 2:**
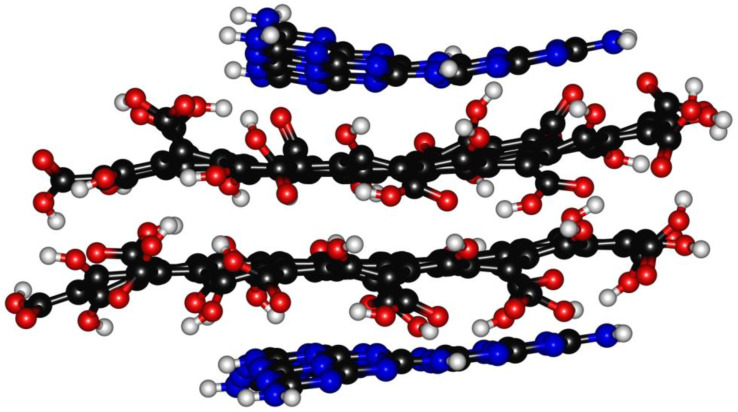
In vacuo optimized CNDs structural model using the PM3-based semi-empirical approach.

The heptazine structure (L_h_^n^) was assembled following the method used by Zhou et al. [54] to produce CNDs composed of pure heptazine. The graphene layer L_g_^n^ could be modeled using at least four geometrical models, as reported by Mandal et al. [19]. We selected type-4 as it showed the lowest energy band gap. According to the Lerf–Klinowski model of graphene oxide [18], oxygen-containing residues symmetrically tailored the L_g_^n^ layer on the edges with oxygen-based functionalities. The oxygen-based functions of the CND edges are still a matter of debate, but Kirbas et al. [29] have reported how they can be tuned by varying the amounts of urea and organic acids used for their production. We selected, as oxygen-based functionalities, hydroxyl and carboxylic groups to evaluate the effects of the presence of a strong network of hydrogen bonds inside the structure. This condition could promote the dismutation of carbonyl groups under harsh synthetic environments while avoiding the presence of carbonyl residues [23]. The other key point to be defined was related to the molecular weight of L_g_^n^. We limited the L_g_^n^ size to 81 carbon atoms in order to achieve a four-layer structure with two each of L_h_^n^ and L_g_^n^, respectively, for an average molecular weight higher than 4000 g/mol but with an expected size of below 3 nm, in accordance with the data reported in the literature regarding the common size of CNDs [55]. The layered structure was optimized in vacuo at a temperature of 303.15 K, and the graphical results are shown in Figure 2.

The optimized structure was characterized by a free energy of 180.4 kcal/mol and a maximum size of 2.0 × 1.3 nm, with different layer distances related to the intrinsic asymmetry of the model used. The L_g_^1^–L_g_^2^ distance was found to be up to 0.41 nm, while the maximum distance between the L_g_^n^ and L_h_^n^ was lower: 0.39–0.34. The L_g_^1^–L_g_^2^ interlayer distances were considerably higher than that of the neat graphite structure (0.335 nm) [56], which is in agreement with the massive functionalization that induced this defectiveness. The presence of functional groups on the edge of the L_g_^n^ induced a deformation due to the simultaneous effects of steric hindrance and the attraction/repulsion between the oxygen-based functions [57,58]. The L_h_^1^ and L_h_^2^ layer was distorted, with dihedral angles of 1.4° and 7.8° due to the interaction between the nitrogen residues and oxygen-based functionalities. Interestingly, L_h_^n^ was non-centered on the L_g_^n^, leaving a more oxygen-rich region on one of the CNDs’ extremities. This was due to the asymmetry of the L_g_^n^, which can promote the favoring of the formation of a hydrogen bonds network on the edges, rather than π−π stacking. 

As a matter of fact, this simple model was a rough approximation of CNDs, neglecting a real distribution of the oxygen and nitrogen functions between L_g_^n^ and L_h_^n^, but it was worth considering it due to the simple approach proposed to introduce the graphene-oxidized core into the CNDs. We discuss the limitations and applications of our approach in the dedicated section below.

### 2.2. Doxo@CNDs Model Structures Modelling

**Figure 3:**
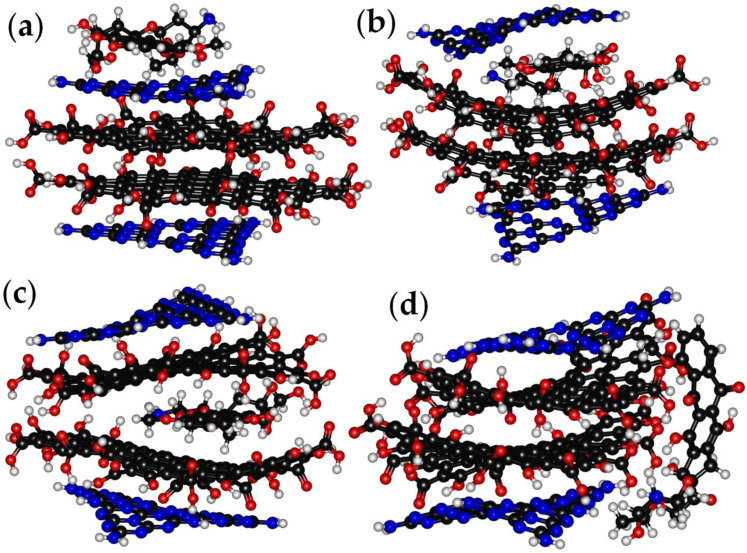
Optimized molecular structure of (**a**) D-CNDs1, (**b**) D-CNDs2, (**c**) D-CNDs3 and (**d**) D-CNDs 3.

The four-layer model adopted to enable us to describe the CNDs allowed several kinds of interaction with Doxorubicin, forming several D-CNDs. We investigated the structures reported in Figure 3, in which Doxorubicin interacted only with L_h_^n^ (Figure 3a, D-CNDs1), intercalated between L_h_^1^ and L_g_^1^ (Figure 3b, D-CNDs2), intercalated between L_g_^1^ and L_g_^2^ (Figure 3c, D-CNDs3) and interacted with all L_h_^n^ and L_g_^n^ (Figure 3d, D-CNDs4) values. In Table 1, the main structural and energetic features in are summarized.

D-CNDs1 showed a consistent increase in terms of L_h_^1^–L_g_^1^ distance compared with CNDs up to 0.55 nm with a decrement of the L_h_^n^ strains and significant increase in L_g_^n^ up to 15.9°. This modification occurred due to the strong interaction of Doxorubicin with L_h_^1^ at a distance of 0.25 nm. As it clearly emerged, the decrement of the L_h_^n^ strain compacted the L_g_^n^, promoting the interactions through weak forces. When the Doxorubicin intercalated the CNDs layers, greater changes occurred in the CNDs’ structure. D-CNDs2 showed an increase in all layers’ distance and the strains. Interestingly, Doxorubicin was closer to L_g_^1^ (0.36 nm) than L_h_^1^ (0.40 nm), probably due to a better interaction with the graphene layer as a consequence of its greater spatial dimension. Similar distances were observed in D-CNDs3, where the distance between the Doxorubicin and L_g_^n^ layer was the same (0.36–0.37 nm). In this case, we observed the maximum strain of L_g_^n^, reaching 63.2° due to the formation of a task-like structure in which Doxorubicin found its place. D-CNDs4 proved that the interaction with all the CNDs layers also induced a significant structural modification, with the increase in L_h_^1^–L_g_^1^ distance being up to 0.42 nm and the layers being distorted. These computational results open an interesting pathway to evaluating the microscopic analysis of D-CNDs in which structural modification could be correlated with the kind of conjugation that has occurred in the specie. Considering the values of E_i_, the more stable structure was D-CNDs3, in which Doxorubicin is fully contained in the CNDs structures and stabilized by the π−π interactions of L_g_^n^ (E_i_ −39.5 kcal/mol), while the D-CNDs2 and D-CNDs4 were less stable (E_i_ 7.1 and 16.6 kcal/mol, respectively). D-CNDs1 was also stabilized by the ordering of the piling-up of Doxorubicin on L_h_^1^ with an E_i_ −28.5 kcal/mol. These results suggest that the π−π interactions were the main driving force for the interaction between the Doxorubicin and CNDs, rather than the hydrogen bond ones.

**Figure 4:**
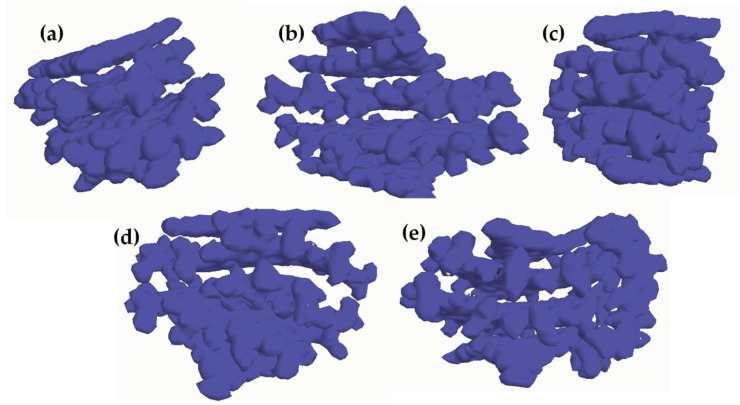
Electron density maps of (**a**) CNDs, (**b**) D-CNDs1, (**c**) D-CNDs2, (**d**) D-CNDs3 and (**e**) D-CNDs 3.

Doxorubicin loading to the CND also affected the HOMO–LUMO gap (Δ_HL_). The calculated Doxorubicin Δ_HL_ was in agreement with the one reported by Lopez-Chavez et al. [59] using DFT approaches. The CNDs showed an Δ_HL_ of up to 1.56 eV, while the presence of Doxorubicin altered the Δ_HL_ by both increasing or decreased it based on its position. D-CNDs1 and D-DCNDs4 (1.38 and 1.46 eV, respectively) showed an Δ_HL_ lower than CNDs, while both D-CNDs2 and D-CNDs3 showed sensible increments of Δ_HL_ (1.71 and 1.58 eV, respectively). As reported by Bharathy et al. [60], a decrease in Δ_HL_ could be correlated to an increase in the reactivity of the systems, and this suggested that Doxorubicin-containing CNDs were less reactive when Doxorubicin was inserted between the layers. Particularly, the insertion between L_h_^1^ and L_g_^1^ seemed to be the best configuration among the ones tested due to the interaction with two layers without a massive alteration, as in the case of D-CNDs4. The insertion between L_g_^1^ and L_g_^2^ was not so effective, probably due to the exposure of a consistent part of Doxorubicin to the external environment. Even if Δ_HL_ is a powerful tool for discussing the stability of a system, it is not entirely sufficient to describe the kind of interaction occurring in the system. According to Johnson et al. [61], the evaluation of interactions could be properly conducted using only NBO analysis to evaluate the atom–atom contribution to the stabilization. Nonetheless, this approach has a very high computational cost for large systems such as the one that is very representative of those used in the interpretation of CDs. Alternatively, it is possible to evaluate the electron density and use electron maps such those reported in Figure 4.

The electron density map showed a rich region between L_g_^1^ and L_g_^2^ in CNDs that was reduced in the D-CNDs, with the exception of D-CNDs2. In this case, the insertion of Doxorubicin between L_h_^1^ and L_g_^1^ induced the concentration of electron density between the reducing L_g_^n^ layers. The localization of high electron density on Doxorubicin in D-CNDs3 and D-CNDs4 suggested that it can react more easily in these two configurations than in the others.

## 3. Computational Methods

The Doxorubicin, CND and D-CND structures were drawn using Chem Sketch (ACD Lab, Toronto, CA, USA), and they were modelled using ArgusLab 4.0.1 [62,63] software without considering the discrete solvent medium. The calculations were performed using a personal computer with the Windows 10 operating system (built 19045) equipped with an Intel(R) Core(TM) i5-10210U CPU @ 1.60 GHz, 2112 Mhz, 4 cores, 8 logic gates and 16 GB of RAM installed. Conformational analysis was carried out through geometrical optimization using the PM3 semi-empirical quantum mechanical parameterization of the Hartree–Fock calculation method.

The geometry of Doxorubicin, CND and D-CND structures were considered optimized when the converged set point of 0.001 kcal/(Åmol) was reached using the Polak–Ribiere conjugate gradient algorithm for the optimization process [64] using up to 50,000 cycles. The interaction energies between the Doxorubicin and CNDs were calculated as follows:

(1) $$E_{i}=E_{D-CNDs}-(E_{CNDs}+E_{D})$$

where *E_i_* is the interaction energy, *E_D-CNDs_* is the total energy of the optimized Doxorubicin–CNDs structure, *E_D-CNDs_* is the total energy of the free optimized CNDs structure and *E_D_* is the total energy of the free optimized Doxorubicin.

The optimized structures were also used for the numerical evaluation of the highest occupied molecular orbital (HOMO) and lower unoccupied molecular orbital (LUMO) energetic values.

We also report the electron density map produced by the calculation ran using ArgusLab 4.0.1 with a 40-slice grid. 

The visualization of the molecules and geometrical parameters of the optimized structures were evaluated using Avogadro 1.2 software [65]. The layer distances are reported as the maximum distance, and the angular strain is defined as the dihedral angle formed by the layers.

## 4. The Interpretation of CDs Structures and Their Simulations: A Critical Discussion

**Figure 5:**
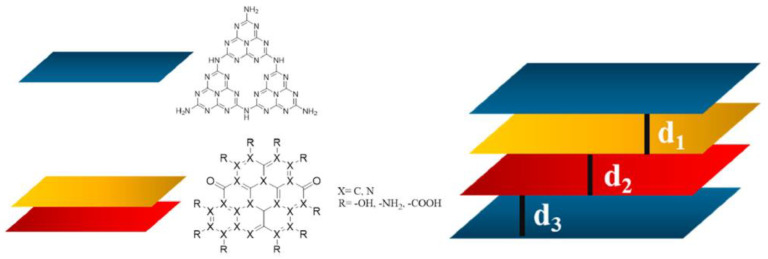
Real data-driven interpretative scheme of CNDs. Reprinted with all permission from Zhou et al. [54].

The use of a computation model for the interpretation of CDs can be a powerful tool, but it should be used with great attention. The first limitation that the computational routes showed is related to the nature of what is simulated. As reported by Zhou et al. [54], the simple synthesis of CNDs led to the production of mixtures of great complexity, even using high-performance purification systems such as dialysis. The authors identified at least five well-characterized species inside the CNDs, with different distributions of functionalities. The authors proposed a data-driven interpretation model in which a layered structure was highly functionalized and variable. Nevertheless, the authors clearly stated that each fraction produced was not composed of a single specie but of a narrow distribution of very similar species. According to this capital finding, the computational approaches to CDs interpretation are intrinsically limited due to the mismatch between the simulated input and real materials. Nonetheless, there is a limited number of cases in which the gap between reality and the simulation is narrow enough to be neglected. GQDs can be investigated using the computation tool together with a complex set of electron microscopy and spectroscopic techniques, allowing for good agreement between the empirical data and computational outputs. On the contrary, PCDs are out of the range of possibility due to their random shapes. CNDs lay in between PCDs and GCDs, showing simple features to be integrated in a simulation such as a heptazine structure, and in more complex ones, distribution functions, as reported in Figure 5.

As clearly emerged, the L_h_^n^ layers have been defined, and their identification can be made using a combination of NMR and mass spectroscopy investigation. The definition of the L_g_^n^ layers is practically impossible and there is no solution to their unique identification. Furthermore, the interaction between L_h_^n^ and L_g_^n^ is only considered to be a weak interaction, but it is impossible to exclude the formation of a chemical bond between them, which could probably form as a consequence of the opening of epoxy functions. Therefore, the trade-off between representability and the reality of CND models should be evaluated considering how the model is able to predict the properties, but also, this approach could be misleading because the distribution of the particles could show the same properties as one of the particles. Nowadays, the computational study of CNDs could provide interesting insight into how these species interact with other molecules, but they are still far from being used as a predictive instrument with which to rationally design the synthesis and applications.

The customizable design of CNDs is of utmost relevance for all of those applications that require the fine tuning of the bot size and chemical functionalities [66]. 

Gao et al. [67] studied the use of the ab initio approach to CND hybrid materials coupled with CDs while assuming a layered structured for species. The authors showed the relevance of both the size and the shape of the layers in driving both of the relative positions and the alignment of bands. The authors proved that a considerably small enlargement size of the CNDs was able to enhance the visible light response of the species, forming a proper type-II van der Waals heterojunction between the CNDs and CDs [68]. The tuning of the band gap is also crucial for all of those catalytic applications, ranging from electrochemical to photochemical ones [69,70].

Li et al. [71] evaluated the effect of CNDs on the expression of protein kinases for the regulation of the cell signaling pathway. The authors showed the crucial role played by the phosphorylation of CNDs and how this is related to the edge functionalities.

The results discussed showed that the cutting edges applications of CNDs required a proper integration of empirical data with a solid interpretation that only a computational approach could provide. Nevertheless, some key issue remain unsolved together with opportunity as summarized in Table 2.

## 5. Conclusions

The computational studies of CDs have made the first steps and already provided key information that have driven the research in new directions. Here, we describe a simple, representative and solid model of CNDs useful for evaluating their interaction with Doxorubicin, a widely used anticancer molecule. The modelled D-CNDs species showed various unique structures, suggesting that the intercalated species are more stable if the intercalation occurred between L_g_^n^ or through adsorption directly onto L_h_^n^. Interestingly, π−π interactions seemed to play a greater role compared to hydrogen bond, but this result is quite sensitive to the model used. Nevertheless, this first computational study on three-dimensional D-CND models enlightens the critical role of the modelling approach in order to fully understand the CDs’ physiochemical behavior. 

In the future, we aim to refine this model using a synthetic approach oriented towards the production of well-defined CNDs with reproducible and uniquely identifiable features. This will allow us to definitively prove the robustness of the approach in the prediction of the CDs’ chemical, optical and geometrical properties.

## Figures and Tables

**Table 1:** Summary of main structural features and energetic outcomes of Doxorubicin, CNDs and D-CNDs species.

Specie	Distances (nm)				Angular Strain (°)			Free Energy (kcal/mol)	E_i_(kcal/mol)	Δ_HL_^e^ (eV)
	L_h_^1^–L_g_^1^	L_g_^1^–L_g_^2^	L_g_^2^–L_h_^2^	Doxorubicin-Layer	L_h_^1^	L_h_^2^	L_g_^n^			
Doxorubicin	--	--	--	--	--	--	--	128.7	--	0.33
CNDs	0.39	0.41	0.34	--	1.4	7.8	9.1	180.4	--	1.56
D-CNDs1	0.55	0.39	0.67	0.25 ^a^	0.3	0.1	15.9	280.6	−28.5	1.38
D-CNDs2	0.70	0.50	0.42	0.40 ^a^/0.36 ^b^	12.3	66.1	26.1	316.2	7.1	1.71
D-CNDs3	0.35	0.73	0.38	0.36 ^b^/0.37 ^c^	0.2	0.1	63.2	269.6	−39.5	1.58
D-CNDs4	0.42	0.36	0.34	0.50 ^d^	21.4	58.2	42.6	325.6	16.5	1.46

^a^ Minimum distance between Doxorubicin and L_h_^1^. ^b^ Minimum distance between Doxorubicin and L_g_^1^. ^c^ Minimum distance between Doxorubicin and L_g_^2^. ^d^ Minimum distance between Doxorubicin and CNDs. ^e^ Δ_HL_ = energy value of LUMO − energy value of HOMO.

**Table 2:** Summary of advantages and unresolved issue related to CNDs modelling for custom-designed applications.

Advantages	Disadvantages
▪ Comprehensive interpretations of the spatial arrangement of the CNDs structures. ▪ Evaluation of band gap. ▪ Description of electrochemical properties. ▪ Model the interaction between CNDs and biological species such as protein and nucleic acids.	▪ Required a great analytic efforts to define the chemical functionalities. ▪ Limitation to GQDs and CNDs. ▪ Required long times. ▪ For several application is necessary only the knowledge of physiochemical properties.

## References

[1] Xu X., Ray R., Gu Y., Ploehn H.J., Gearheart L., Raker K., Scrivens W.A. (2004). Electrophoretic analysis and purification of fluorescent single-walled carbon nanotube fragments. J. Am. Chem. Soc..

[2] Giordano M.G., Seganti G., Bartoli M., Tagliaferro A. (2023). An Overview on Carbon Quantum Dots Optical and Chemical Features. Molecules.

[3] Yan F., Sun Z., Zhang H., Sun X., Jiang Y., Bai Z. (2019). The fluorescence mechanism of carbon dots, and methods for tuning their emission color: A review. Microchim. Acta.

[4] Connerade J.P. (2009). A review of quantum confinement. AIP Conference Proceedings.

[5] Yan X., Cui X., Li B., Li L.-S. (2010). Large, solution-processable graphene quantum dots as light absorbers for photovoltaics. Nano Lett..

[6] Zhu L., Shen D., Wu C., Gu S. (2020). State-of-the-Art on the Preparation, Modification, and Application of Biomass-Derived Carbon Quantum Dots. Ind. Eng. Chem. Res..

[7] Chen B.-B., Liu M.-L., Gao Y.-T., Chang S., Qian R.-C., Li D.-W. (2023). Design and applications of carbon dots-based ratiometric fluorescent probes: A review. Nano Res..

[8] Gallareta-Olivares G., Rivas-Sanchez A., Cruz-Cruz A., Hussain S.M., González-González R.B., Cárdenas-Alcaide M.F., Iqbal H.M., Parra-Saldívar R. (2023). Metal-doped carbon dots as robust nanomaterials for the monitoring and degradation of water pollutants. Chemosphere.

[9] Lo Bello G., Bartoli M., Giorcelli M., Rovere M., Tagliaferro A. (2022). A Review on the Use of Biochar Derived Carbon Quantum Dots Production for Sensing Applications. Chemosensors.

[10] Jing H.H., Bardakci F., Akgöl S., Kusat K., Adnan M., Alam M.J., Gupta R., Sahreen S., Chen Y., Gopinath S.C. (2023). Green Carbon Dots: Synthesis, Characterization, Properties and Biomedical Applications. J. Funct. Biomater..

[11] Hui S. (2023). Carbon dots (CDs): Basics, recent potential biomedical applications, challenges, and future perspectives. J. Nanopart. Res..

[12] Sendão R.M., Esteves da Silva J.C., Pinto da Silva L. (2023). Applications of Fluorescent Carbon Dots as Photocatalysts: A Review. Catalysts.

[13] Sun P., Xing Z., Li Z., Zhou W. (2023). Recent Advances in Quantum Dots Photocatalysts. Chem. Eng. J..

[14] Sikiru S., Oladosu T.L., Kolawole S.Y., Mubarak L.A., Soleimani H., Afolabi L.O., Toyin A.-O.O. (2023). Advance and prospect of carbon quantum dots synthesis for energy conversion and storage application: A comprehensive review. J. Energy Storage.

[15] Zhai Y., Zhang B., Shi R., Zhang S., Liu Y., Wang B., Zhang K., Waterhouse G.I., Zhang T., Lu S. (2022). Carbon dots as new building blocks for electrochemical energy storage and electrocatalysis. Adv. Energy Mater..

[16] Georgakilas V., Perman J.A., Tucek J., Zboril R. (2015). Broad family of carbon nanoallotropes: Classification, chemistry, and applications of fullerenes, carbon dots, nanotubes, graphene, nanodiamonds, and combined superstructures. Chem. Rev..

[17] Mansuriya B.D., Altintas Z. (2021). Carbon Dots: Classification, properties, synthesis, characterization, and applications in health care—An updated review (2018–2021). Nanomaterials.

[18] Lerf A., He H., Forster M., Klinowski J. (1998). Structure of graphite oxide revisited. J. Phys. Chem. B.

[19] Mandal B., Sarkar S., Sarkar P. (2012). Exploring the electronic structure of graphene quantum dots. J. Nanopart. Res..

[20] Yan X., Li B., Cui X., Wei Q., Tajima K., Li L.-s. (2011). Independent tuning of the band gap and redox potential of graphene quantum dots. J. Phys. Chem. Lett..

[21] Yan X., Cui X., Li L.-s. (2010). Synthesis of large, stable colloidal graphene quantum dots with tunable size. J. Am. Chem. Soc..

[22] Hai X., Mao Q.-X., Wang W.-J., Wang X.-F., Chen X.-W., Wang J.-H. (2015). An acid-free microwave approach to prepare highly luminescent boron-doped graphene quantum dots for cell imaging. J. Mater. Chem. B.

[23] Seven E.S., Kirbas Cilingir E., Bartoli M., Zhou Y., Sampson R., Shi W., Peng Z., Ram Pandey R., Chusuei C.C., Tagliaferro A. (2023). Hydrothermal vs microwave nanoarchitechtonics of carbon dots significantly affects the structure, physicochemical properties, and anti-cancer activity against a specific neuroblastoma cell line. J. Colloid Interface Sci..

[24] Xia C., Zhu S., Feng T., Yang M., Yang B. (2019). Evolution and synthesis of carbon dots: From carbon dots to carbonized polymer dots. Adv. Sci..

[25] Chen J., Li F., Gu J., Zhang X., Bartoli M., Domena J.B., Zhou Y., Zhang W., Paulino V., Ferreira B.C. (2023). Cancer cells inhibition by cationic carbon dots targeting the cellular nucleus. J. Colloid Interface Sci..

[26] Zhang W., Chen J., Gu J., Bartoli M., Domena J.B., Zhou Y., Ferreira B.C., Kirbas Cilingir E., McGee C.M., Sampson R. (2023). Nano-carrier for gene delivery and bioimaging based on pentaetheylenehexamine modified carbon dots. J. Colloid Interface Sci..

[27] Liyanage P.Y., Graham R.M., Pandey R.R., Chusuei C.C., Mintz K.J., Zhou Y., Harper J.K., Wu W., Wikramanayake A.H., Vanni S. (2019). Carbon Nitride Dots: A Selective Bioimaging Nanomaterial. Bioconjugate Chem..

[28] Mintz K.J., Bartoli M., Rovere M., Zhou Y., Hettiarachchi S.D., Paudyal S., Chen J., Domena J.B., Liyanage P.Y., Sampson R. (2021). A deep investigation into the structure of carbon dots. Carbon.

[29] Kirbas E.C., Sankaran M., Garber J.M., Vallejo F.A., Bartoli M., Tagliaferro A., Vanni S., Graham R., Leblanc R.M. (2022). Surface Modification Nanoarchitectonics of Carbon Nitride Dots for Better Drug Loading and Higher Cancer Selectivity. Nanoscale.

[30] Wiśniewski M. (2022). The Consequences of Water Interactions with Nitrogen-Containing Carbonaceous Quantum Dots—The Mechanistic Studies. Int. J. Mol. Sci..

[31] Liyanage P.Y., Zhou Y., Al-Youbi A.O., Bashammakh A.S., El-Shahawi M.S., Vanni S., Graham R.M., Leblanc R.M. (2020). Pediatric glioblastoma target-specific efficient delivery of gemcitabine across the blood–brain barrier via carbon nitride dots. Nanoscale.

[32] Carvalho C., Santos R.X., Cardoso S., Correia S., Oliveira P.J., Santos M.S., Moreira P.I. (2009). Doxorubicin: The good, the bad and the ugly effect. Curr. Med. Chem..

[33] Rivankar S. (2014). An overview of doxorubicin formulations in cancer therapy. J. Cancer Res. Ther..

[34] Zhang W., Dang G., Dong J., Li Y., Jiao P., Yang M., Zou X., Cao Y., Ji H., Dong L. (2021). A multifunctional nanoplatform based on graphitic carbon nitride quantum dots for imaging-guided and tumor-targeted chemo-photodynamic combination therapy. Colloids Surf. B Biointerfaces.

[35] Dong J., Zhao Y., Chen H., Liu L., Zhang W., Sun B., Yang M., Wang Y., Dong L. (2018). Fabrication of PEGylated graphitic carbon nitride quantum dots as traceable, pH-sensitive drug delivery systems. New J. Chem..

[36] Dong J., Zhao Y., Wang K., Chen H., Liu L., Sun B., Yang M., Sun L., Wang Y., Yu X. (2018). Fabrication of graphitic carbon nitride quantum dots and their application for simultaneous fluorescence imaging and pH-responsive drug release. ChemistrySelect.

[37] Rashid A., Perveen M., Khera R.A., Asif K., Munir I., Noreen L., Nazir S., Iqbal J. (2021). A DFT study of graphitic carbon nitride as drug delivery carrier for flutamide (anticancer drug). J. Comput. Biophys. Chem..

[38] Zaboli A., Raissi H., Farzad F. (2021). Molecular interpretation of the carbon nitride performance as a template for the transport of anti-cancer drug into the biological membrane. Sci. Rep..

[39] Shaki H., Raissi H., Mollania F., Hashemzadeh H. (2020). Modeling the interaction between anti-cancer drug penicillamine and pristine and functionalized carbon nanotubes for medical applications: Density functional theory investigation and a molecular dynamics simulation. J. Biomol. Struct. Dyn..

[40] Valatin J. (1961). Generalized hartree-fock method. Phys. Rev..

[41] Bartlett R.J., Stanton J.F. (1994). Applications of Post-Hartree—Fock Methods: A Tutorial. Rev. Comput. Chem..

[42] Dewar M.J., Thiel W. (1977). Ground states of molecules. 38. The MNDO method. Approximations and parameters. J. Am. Chem. Soc..

[43] Dewar M.J., Thiel W. (1977). A semiempirical model for the two-center repulsion integrals in the NDDO approximation. Theor. Chim. Acta.

[44] Engelke R. (1981). Limitations on mndo and mndo/ci computations of activation barriers. Chem. Phys. Lett..

[45] Dewar M.J., Zoebisch E.G., Healy E.F., Stewart J.J. (1985). Development and use of quantum mechanical molecular models. 76. AM1: A new general purpose quantum mechanical molecular model. J. Am. Chem. Soc..

[46] Stewart J.J. (1989). Optimization of parameters for semiempirical methods II. Applications. J. Comput. Chem..

[47] Cavasotto C.N., Aucar M.G., Adler N.S. (2019). Computational chemistry in drug lead discovery and design. Int. J. Quantum Chem..

[48] Repasky M.P., Chandrasekhar J., Jorgensen W.L. (2002). PDDG/PM3 and PDDG/MNDO: Improved semiempirical methods. J. Comput. Chem..

[49] Toledo M.V., Briand L.E., Ferreira M.L. (2022). A Simple Molecular Model to Study the Substrate Diffusion into the Active Site of a Lipase-Catalyzed Esterification of Ibuprofen and Ketoprofen with Glycerol. Top. Catal..

[50] Bystrov V., Paramonova E., Meng X., Shen H., Wang J., Fridkin V. (2022). Polarization switching in nanoscale ferroelectric composites containing PVDF polymer film and graphene layers. Ferroelectrics.

[51] Stewart J.J. (1991). Optimization of parameters for semiempirical methods. III extension of pm3 to be, mg, zn, ga, ge, as, se, cd, in, sn, sb, te, hg, tl, pb, and bi. J. Comput. Chem..

[52] Ignatov S., Razuvaev A., Kokorev V., Alexandrov Y.A. (1996). Extension of the PM3 Method on s, p, d Basis. Test Calculations on Organochromium Compounds. J. Phys. Chem..

[53] Rono N., Kibet J.K., Martincigh B.S., Nyamori V.O. (2021). A review of the current status of graphitic carbon nitride. Crit. Rev. Solid State Mater. Sci..

[54] Zhou Y., Kandel N., Bartoli M., Serafim L.F., ElMetwally A.E., Falkenberg S.M., Paredes X.E., Nelson C.J., Smith N., Padovano E. (2022). Structure-activity relationship of carbon nitride dots in inhibiting Tau aggregation. Carbon.

[55] Liu H., Wang X., Wang H., Nie R. (2019). Synthesis and biomedical applications of graphitic carbon nitride quantum dots. J. Mater. Chem. B.

[56] Lipson H.S., Stokes A. (1942). The structure of graphite. Proc. R. Soc. London. Ser. A. Math. Phys. Sci..

[57] Sakorikar T., Vayalamkuzhi P., Jaiswal M. (2020). Geometry dependent performance limits of stretchable reduced graphene oxide interconnects: The role of wrinkles. Carbon.

[58] Gómez-Navarro C., Burghard M., Kern K. (2008). Elastic properties of chemically derived single graphene sheets. Nano Lett..

[59] Lopez-Chavez E., Garcia-Quiroz A., Santiago-Jiménez J.C., Díaz-Góngora J.A., Díaz-López R., de Landa Castillo-Alvarado F. (2021). Quantum–mechanical characterization of the doxorubicin molecule to improve its anticancer functions. MRS Adv..

[60] Bharathy G., Prasana J.C., Muthu S. (2018). Molecular conformational analysis, vibrational spectra, NBO, HOMO–LUMO and molecular docking of modafinil based on density functional theory. Int. J. Cur. Res. Rev.

[61] Johnson E.R., Keinan S., Mori-Sánchez P., Contreras-García J., Cohen A.J., Yang W. (2010). Revealing Noncovalent Interactions. J. Am. Chem. Soc..

[62] Chaudhary N.K., Mishra P. (2017). Metal complexes of a novel Schiff base based on penicillin: Characterization, molecular modeling, and antibacterial activity study. Bioinorg. Chem. Appl..

[63] Agrahari A.K. (2017). A computational approach to identify a potential alternative drug with its positive impact toward PMP22. J. Cell. Biochem..

[64] Zhang L., Zhou W., Li D.-H. (2006). A descent modified Polak–Ribière–Polyak conjugate gradient method and its global convergence. IMA J. Numer. Anal..

[65] Hanwell M.D., Curtis D.E., Lonie D.C., Vandermeersch T., Zurek E., Hutchison G.R. (2012). Avogadro: An advanced semantic chemical editor, visualization, and analysis platform. J. Cheminform..

[66] Liu S., Li D., Sun H., Ang H.M., Tadé M.O., Wang S. (2016). Oxygen functional groups in graphitic carbon nitride for enhanced photocatalysis. J. Colloid Interface Sci..

[67] Gao G., Jiao Y., Ma F., Jiao Y., Waclawik E., Du A. (2015). Carbon nanodot decorated graphitic carbon nitride: New insights into the enhanced photocatalytic water splitting from ab initio studies. Phys. Chem. Chem. Phys..

[68] Jin C., Ma E.Y., Karni O., Regan E.C., Wang F., Heinz T.F. (2018). Ultrafast dynamics in van der Waals heterostructures. Nat. Nanotechnol..

[69] Feng J., Liu G., Yuan S., Ma Y. (2017). Influence of functional groups on water splitting in carbon nanodot and graphitic carbon nitride composites: A theoretical mechanism study. Phys. Chem. Chem. Phys..

[70] Gao Y., Hou F., Hu S., Wu B., Wang Y., Zhang H., Jiang B., Fu H. (2018). Graphene quantum-dot-modified hexagonal tubular carbon nitride for visible-light photocatalytic hydrogen evolution. ChemCatChem.

[71] Li X., Zhou Y., Xu Y., Xu H., Wang M., Yin H., Ai S. (2016). A novel photoelectrochemical biosensor for protein kinase activity assay based on phosphorylated graphite-like carbon nitride. Anal. Chim. Acta.

